# Expansion of Pathogen-Specific Mono- and Multifunctional Th1 and Th17 Cells in Multi-Focal Tuberculous Lymphadenitis

**DOI:** 10.1371/journal.pone.0057123

**Published:** 2013-02-25

**Authors:** Nathella Pavan Kumar, Rathinam Sridhar, Vaithilingam V. Banurekha, Dina Nair, Mohideen S. Jawahar, Thomas B. Nutman, Subash Babu

**Affiliations:** 1 National Institutes of Health–International Center for Excellence in Research, Chennai, India; 2 Government Stanley Medical Hospital, Chennai, India; 3 National Institute for Research in Tuberculosis, Chennai, India; 4 Laboratory of Parasitic Diseases, National Institute of Allergy and Infectious Diseases, National Institutes of Health, Bethesda, Maryland, United States of America; University of Delhi, India

## Abstract

**Background:**

Th1 and Th17 responses are known to play an important role in immunity to pulmonary tuberculosis (PTB), although little is known about their role in extrapulmonary forms of tuberculosis (TB).

**Methods:**

To identify the role of Th1, Th17, and Th22 cells in multi-focal TB lymphadenitis (TBL), we examined mycobacteria–specific immune responses in the whole blood of individuals with PTB (*n* = 20) and compared them with those with TBL (*n* = 25).

**Results:**

Elevated frequencies of CD4^+^ T cells expressing IFN- γ, TNF-α, and IL-2 were present in individuals with TBL compared with those with PTB at baseline and in response to ESAT-6 and CFP-10. Similarly, increased frequencies of CD4^+^ T cells expressing IL-17A, IL-17F, and IFN-γ were also present in individuals with TBL at baseline and following ESAT-6 and CFP-10 stimulation although no significant difference in frequency of Th22 cells was observed. Finally, frequencies of Th1 (but not Th17) cells exhibited a significantly negative correlation with natural regulatory T cell frequencies at baseline.

**Conclusions:**

Multi-focal TB lymphadenitis is therefore characterized by elevated frequencies of Th1 and Th17 cells, indicating that Th1 and Th17 responses in TB disease are probably correlates of disease severity rather than of protective immunity.

## Introduction

Tuberculosis (TB) is a spectral disease with host responses controlling disease severity and extrapulmonary dissemination. Although *Mycobacterium tuberculosis* (Mtb) infects ∼ 2 billion people worldwide, 90% of Mtb-infected individuals are able to resist overt disease (active TB) development and manifest only latent infection [1]; the mechanism by which these individuals resist development of active disease is still not clear. In addition, how Mtb-infected individuals become more susceptible to extrapulmonary dissemination following initial infection is also not known. Extrapulmonary TB is a significant health problem worldwide because of the difficulties of diagnosis and treatment. Tuberculous lymphadenitis (TBL) is the most common presentation of extrapulmonary TB, accounting for 30–40% of cases [2]. Multi-focal TBL is thought to reflect extrapulmonary spread by the hematogenous route from an initial focus in the lung and, as such, represents a more disseminated form of active TB disease [3].

T cell differentiation into Th1 and Th2 lineages underlies the pathogenesis of a variety of infectious and allergic diseases [4]. With the advent of newer techniques, a variety of T cell subsets has been identified, including regulatory T cells (Tregs), Th17 cells, Th22 cells, and multifunctional T cells, among others [5], [6], [7], [8]. Based on murine models as well as some human data, immunity to Mtb requires Th1 responses and (to a lesser extent) Th17 responses [9], [10]. Thus, IL-12, IFN-γ, and TNF-α–along with IL-17 and IL-23–all play important roles in the induction and maintenance of protective immune responses against tuberculous disease [11], [12], [13], [14], [15], [16]. Similar to Th1 and Th17 cells, Th22 cells are thought to be potentially involved in protection against TB infection [17], although their exact role remains to be elucidated. Further, multifunctional T cells, defined by their ability to co-express two or more cytokines, have been associated with resistance to infection in animal models [18]. Thus, while some studies have implicated multifunctional Th1 cells in protective immunity against pulmonary disease [19], [20], other studies have shown that multifunctional Th1 cells might merely reflect the presence of active disease [21], [22]. Although different Th17 subsets have been described recently [23], [24], their role in TB is not known. Finally, very few studies have actually explored the role of Th1, Th17, and Th22 cells in extra-pulmonary TB [25], [26], [27].

To study the role of Th1, Th17, and Th22 cell in the pathogenesis of TBL, we examined baseline, antigen-specific, and polyclonal induction of Th1, Th17 and Th22 cells in TBL and compared them to those in PTB individuals. We show that TBL individuals have elevated frequencies of single, double, and triple cytokine-producing CD4^+^ Th1 and Th17 cells, both at baseline and following mycobacterial antigen stimulation, in comparison with PTB patients. We also show that frequencies of natural Tregs (nTregs) in individuals with TB disease were inversely related to frequencies of mono- and multifunctional Th1 but not Th17 cells. Thus, our data demonstrate that multifunctional T cells in TB disease are an important indicator of disease severity and not necessarily associated with protection against extrapulmonary dissemination.

## Methods

### Study Population

We studied a group of 45 individuals with TB–20 with PTB and 25 with multi-focal TBL (Table 1). Individuals with PTB were diagnosed on the basis of being positive for 3 criteria: (1) positive clinical symptoms and (2) positive radiological finding on chest X-ray and (3) sputum acid-fast bacillus (AFB) Ziehl Neelsen staining. Individuals with multi-focal TBL were diagnosed on the basis of clinical examination showing the presence of multiple foci of lymphadenitis as well as fine needle aspiration cytology and direct microscopic screening for AFB. All individuals were HIV negative and did not differ significantly in age or gender distribution and blood was collected prior to commencement of anti-TB treatment. The individuals were not on any steroid treatment and consecutive samples were collected but the BCG or tuberculin skin test status was not known. This study was specifically approved by the Institutional Review Board of the National Institute of Research in Tuberculosis and informed written consent was obtained from all participants.

**Table 1:** Study population.

Study Demographics	PTB	TBL
No. of subjects recruited	20	25
Gender (M/F)	11/9	8/17
Median Age (Range)	42 (19–64)	35 (18–45)
Smear Grade (0/1+/2+/3+)	0/2/5/13	–
TB Lymphadenitis	–	25
**Immunological profile**	**PTB**	**TBL**
Absolute count of CD4+ T cells	764.7 (291–1603)	839.7 (402–1555)
Frequency CD4+ of Naïve T cells	22.21 (4.15–73.8)	20.79 (4.7–57.4)
Frequency CD4+ of Effector T cells	29.55 (5.87–69.6)	31.13 (11.12–79.4)
Frequency CD4+ of Central memoryT cells	29.73 (11.9–74.6)	28.86 (10.5–82.6)

### *Ex vivo* Analysis

All antibodies used in the study were from BD Bioscience (San José, CA), BD Pharmingen™ (San Diego, CA), eBioscience (San Diego, CA), or R&D Systems (Minneapolis, MN). Absolute numbers of CD4^+^ T cells were enumerated in whole blood using BD Multiset™ 6-Color TBNK cocktail (BD Biosciences). Naïve and memory T cell phenotyping was performed using CD45RA and CCR7 staining on CD4^+^ T cells. Naïve cells were classified as CD45RA^+^CCR7^+^; effector memory cells as CD45RA^–^CCR7^–^; and central memory cells as CD45RA^–^CCR7^+^; and nTregs as CD4^+^CD25^+^Foxp3^+^CD127^dim^. *Ex vivo* intracellular staining for Ki-67 expression on CD4^+^ T cells was performed to determine recent activation/proliferation.

### Antigens

Mycobacterial antigens–early secreted antigen-6 (ESAT-6) and culture filtrate protein-10 (CFP–10) (both from Fitzgerald Industries Intl. Inc, Acton, MA)–were used as the antigenic stimuli, and anti-CD3 antibody was used as the positive control. Final concentrations were 10 µg/ml for ESAT-6 and CFP-10 and 5 µg/ml for anti-CD3.

### *In Vitro* Culture

Whole blood cell cultures were performed to determine the intracellular levels of cytokines. Briefly, whole blood was diluted 1∶1 with RPMI-1640 medium supplemented with penicillin/streptomycin (100 U/100 mg/ml), L-glutamine (2 mM), and HEPES (10 mM) (all from Invitrogen, San Diego, CA) and distributed in 12-well tissue culture plates (Costar, Corning Inc., Corning, NY). The cultures were then stimulated with ESAT-6, CFP-10, or anti-CD3 or media alone in the presence of the costimulatory molecules CD49d/CD28 at 37°C for 6 h. FastImmune™ brefeldin A solution (10 µg/ml) was added after 2 h. After 6 h, centrifugation, washing, and red blood cell lysis were performed. Cells were fixed and cryopreserved at –80°C.

### Intracellular Cytokine Staining

The cells were thawed, washed, and then stained with surface antibodies for 30–60 min. Surface antibodies used were CD3, CD4, and CD8. The cells were washed and permeabilized with BD Perm/Wash™ buffer (BD Biosciences) and stained with intracellular cytokines for an additional 30 min before washing and acquisition. Cytokine antibodies used were IFN-γ, TNF-α, IL-2, IL-17A, IL-17F, and IL-22. Eight-color flow cytometry was performed on a FACSCanto II flow cytometer with FACSDiva software v.6 (Becton Dickinson and Company, Cockeysville, MD). Lymphocyte gating was set by forward and side scatter, and 100,000 lymphocyte events were acquired. Gating for CD4^+^ T cells expressing cytokines was determined by FMO. Data were collected and analyzed using Flow Jo software (TreeStar Inc., Ashland, OR). All data are depicted as frequency of CD4^+^ T cells expressing cytokine(s). Baseline values following media stimulation are depicted as baseline frequency, while frequencies following stimulation with antigens or anti-CD3 are depicted as net frequencies (with baseline values subtracted).

### Statistical Analysis

Data analyses were performed using GraphPad PRISM (GraphPad Software, Inc., San Diego, CA). Geometric means (GM) were used for measurements of central tendency. Statistically significant differences between two groups were analyzed using the nonparametric Mann-Whitney U test. Multiple comparisons were corrected using the Holm’s correction. Correlations were calculated by the Spearman rank correlation test.

## Results

### TBL is Associated with Increased Frequencies of Baseline as Well as Antigen-Specific Mono- and Multifunctional CD4^+^ Th1 Cells

**Figure 1:**
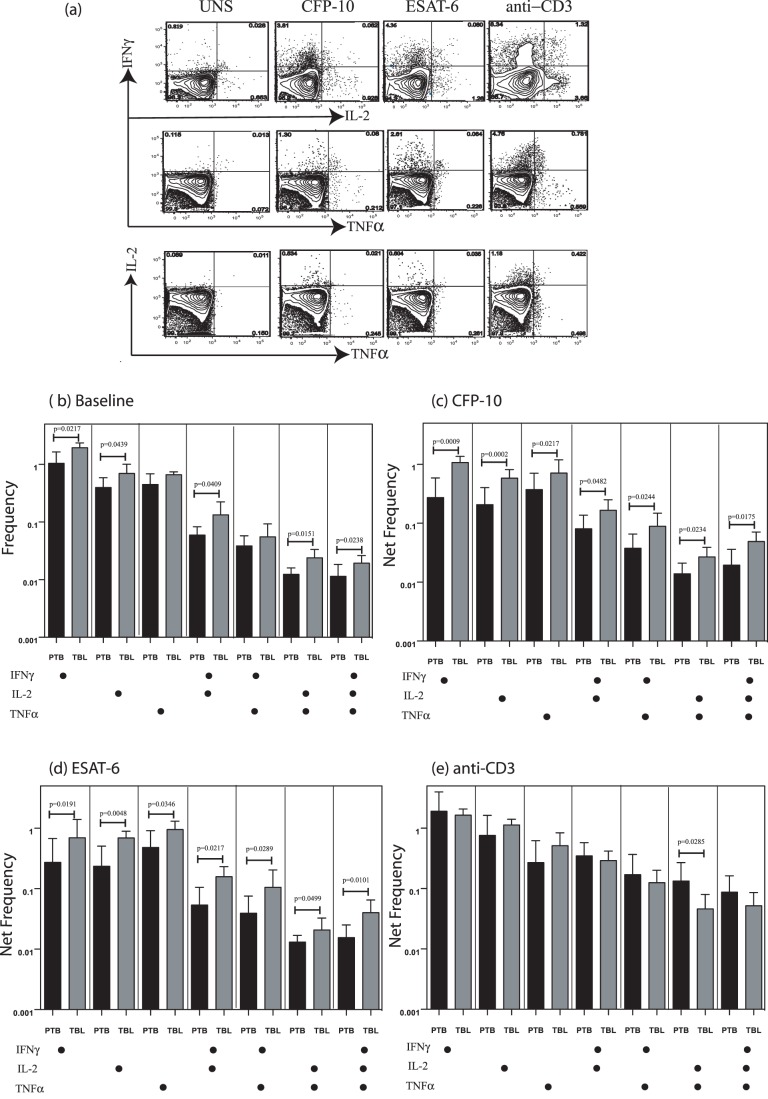
Elevated baseline and antigen-specific frequencies of Th1 cells in tuberculous lymphadenitis (TBL). (*A*) Representative whole-blood intracellular cytokine assay flow data from a TBL individual showing expression of IFN-γ, IL-2, and TNF-α. The plots shown are gated on CD3^+^CD4^+^ T cells. (*B*) Baseline frequency of CD4^+^ T cells expressing one, two, or three cytokines (IFN-γ, IL-2, TNF-α) is shown as bar graphs. The bar represents the geometric mean of the frequency of CD4^+^ T cells expressing the respective cytokine(s), and the error bar represents the 95% confidence interval in TBL (*n* = 25; grey bar) and PTB (*n* = 20; black bar) individuals. (*C, D*) Net frequency of CD4^+^ T cells expressing one, two, or three cytokines in response to CFP-10 (*C*) and ESAT-6 (*D*) is shown in TBL and pulmonary TB (PTB) individuals. (*E*) Net frequency of CD4^+^ T cells expressing the different cytokines in response to anti-CD3 stimulation is shown in TBL and PTB individuals. *P* values were calculated using the Mann-Whitney test.

CD4^+^ T cells play a key role in immune control of Mtb infection, and multifunctional cytokine production of antigen-specific Th1 cells has been felt to be associated with control of infection [18], [28]. To determine the role of multifunctional Th1 cells in TBL, we used multicolor flow cytometry to define the frequencies of baseline, mycobacterial antigen-specific, and polyclonal CD4^+^ T cells expressing IFN-γ, IL-2, and TNF-α (Figure 1A). As shown in Figure 1B, there were significantly elevated frequencies of CD4^+^ T cells expressing either only one cytokine (IFN-γ^+^ or IL-2^+^); two cytokines (IFN-γ^+^IL-2^+^ or IL-2^+^TNF-α^+^); or three cytokines (IFN-γ^+^TNF-α^+^IL-2^+^)at baseline in TBL compared with PTB patients. Similarly, in response to both CFP-10 (Figure 1C) and ESAT-6 (Figure 1D), we observed significantly elevated net frequencies of CD4^+^ T cells expressing either only single cytokine (IFN-γ or IL-2 or TNF-α); or all combinations of two cytokines (IFN-γ^+^IL-2^+^, or IFN-γ^+^TNF-α^+^, or IL-2^+^TNF-α^+^), or all three cytokines (IFN-γ^+^TNF-α^+^IL-2^+^) in TBL compared with PTB patients. Finally, stimulation with anti-CD3 did not induce significant differences in the net frequencies of Th1 responses (with the exception of CD4^+^ T cells expressing IL-2 and TNF-α, which was higher in PTB) between TBL and PTB patients (Figure 1E), indicating that the increased frequency of Th1 cells induced in TBL patients was pathogen specific.

### TBL is Associated with Increased Frequencies of Baseline as Well as Antigen-Specific Mono- and Multifunctional CD4^+^ Th17 Cells

**Figure 2:**
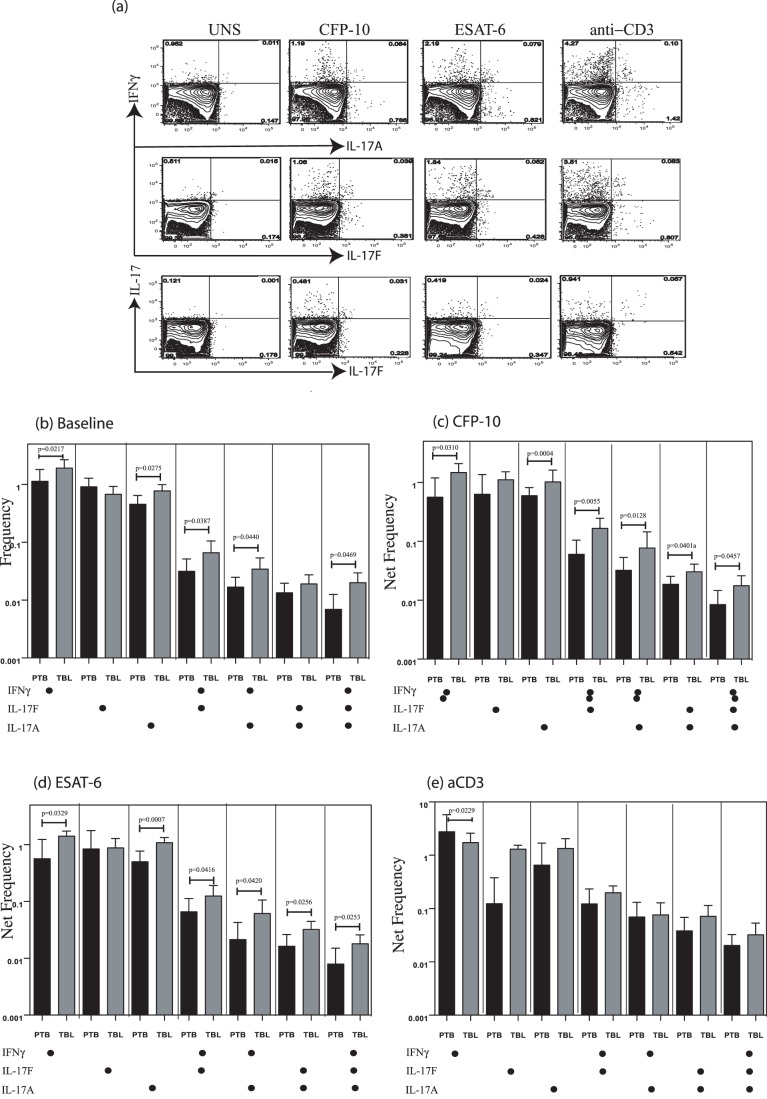
Elevated baseline and antigen-specific frequencies of Th17 cells in tuberculous lymphadenitis (TBL). (*A*) Representative whole-blood intracellular cytokine assay flow data from a TBL individual showing expression of IL-17A, IL-17F and IFN-γ. The plots shown are gated on CD3^+^CD4^+^ T cells. (*B*) Baseline frequency of CD4^+^ T cells expressing one, two, or three cytokines (IL-17A, IL-17F, and IFN-γ) is shown as bar graphs. The bar represents the geometric mean of the frequency of CD4^+^ T cells expressing the respective cytokine(s), and the error bar representing the 95% confidence interval in TBL (*n* = 25; grey bar) and pulmonary TB (PTB; black bar) (*n* = 20) individuals. (*C, D*) Net frequency of CD4^+^ T cells expressing one, two, or three cytokines in response to CFP-10 (*C*) and ESAT-6 (*D*) is shown in TBL and PTB individuals. (*E*) Net frequency of CD4^+^ T cells expressing the different cytokines in response to anti-CD3 stimulation is shown in TBL and PTB individuals. *P* values were calculated using the Mann-Whitney test.

CD4^+^ Th17 cells are thought to play an important role in memory responses to TB infection, but IL-17 is also thought to contribute to pathology [29]. In addition, although different subsets of Th17 cells have been described [24], their role(s) in TB have not been elucidated. To determine the role of Th17 cells in TBL and to identify a multifunctional profile in cytokine production in the Th17 compartment, we used multicolor flow cytometry to define the frequencies of baseline, mycobacterial antigen-specific, and polyclonal CD4^+^ T cells expressing IL-17A, IL-17F, and IFN-γ (Figure 2A). As shown in Figure 2B, there were significantly elevated frequencies of CD4^+^ T cells expressing either only one cytokine (IFN-γ^+^ or IL-17A^+^) or two cytokines (IFN-γ^+^IL-17F^+^ or IFN-γ^+^IL-17A^+^) or three cytokines (IFN-γ^+^IL-17F^+^IL-17A^+^) at baseline in TBL compared with PTB patients. Similarly, in response to both CFP-10 (Figure 2C) and ESAT-6 (Figure 2D), we observed significantly elevated net frequencies of CD4^+^ T cells expressing either only single cytokines (IFN-γ or IL-17A but not IL-17F) or all combinations of two cytokines (IFN-γ^+^IL-17A^+^ or IFN-γ^+^IL-17F^+^ or IL-17A^+^IL-17F^+^) or all three cytokines (IFN-γ^+^IL-17A^+^IL-17F^+^) in TBL compared with PTB patients. Finally, stimulation with anti-CD3 did not induce significant differences in the net frequencies of Th17 responses (with the exception of CD4^+^ T cells expressing IFN-γ alone, which was higher in PTB) between TBL and PTB patients (Figure 2E), indicating that the increased frequency of Th17 cells induced in TBL patients was pathogen specific.

### TBL is Not Associated with Altered Frequencies of Th22 Cells but is Associated with Increased Antigen-Specific Frequencies of IL-22^+^, IL-17A and IL-22^+^, IL-17A^+^, IFN-γ^+^ CD4^+^ T Cells

**Figure 3:**
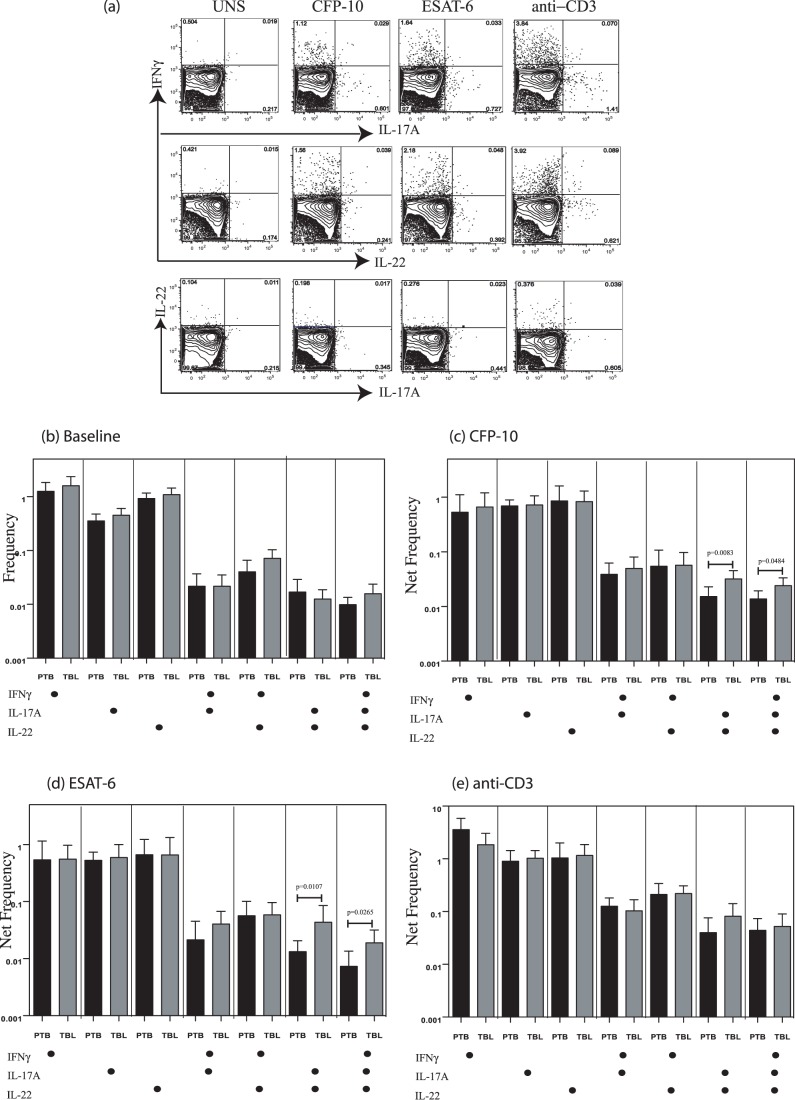
Elevated frequencies of IL-22^+^IL-17A^+^ and IL-22^+^IL-17A^+^IFN-γ^+^ CD4^+^ T cells but not conventional Th22 cells in tuberculous lymphadenitis (TBL). (*A*) Representative whole-blood intracellular cytokine assay flow data from a TBL individual showing expression of IL-22, IL-17A, and IFN-γ. The plots shown are gated on CD3^+^CD4^+^ T cells. (*B*) Baseline frequency of CD4^+^ T cells expressing one, two, or three cytokines (IL-22, IL-17A, and IFN-γ) is shown as bar graphs. The bar represents the geometric mean of the frequency of CD4^+^ T cells expressing the respective cytokine(s), and the error bar represents the 95% confidence interval in TBL (*n* = 24; grey bar) and pulmonary TB (PTB) (*n* = 18; black bar) individuals. (*C, D*) Net frequency of CD4^+^ T cells expressing one, two, or three cytokines in response to CFP-10 (*C*) and ESAT-6 (*D*) is shown in TBL and PTB individuals. (*E*) Net frequency of CD4^+^ T cells expressing the different cytokines in response to anti-CD3 stimulation is shown in TBL and PTB individuals. *P* values were calculated using the Mann-Whitney test.

The role of Th22 cells in TB infection has not been well characterized. To characterize the role of Th22 cells, we used a panel consisting of IL-22, IL-17A, and IFN-γ. We reasoned that this would enable us to better define Th22 cells (IL-22^+^IL-17A^–^IFN-γ^–^) and also examined the co-expression of Th17 (IL-17A) and Th1 (IFN-γ) cytokines. As shown in Figure 3A, TB antigens and anti-CD3 induced the expression of any or all three cytokines in CD4^+^ T cells; however, examination of Th22 cells failed to reveal any significant alteration in the frequency of this population (IL-22^+^IL-17A^–^IFN-γ^–^) in TBL patients compared with PTB individuals at baseline or following antigen or anti-CD3 stimulation (Figure 3, B–E). Both CFP-10 (Figure 3C) and ESAT-6 (Figure 3D) induced significantly increased frequencies of CD4^+^ T cells expressing IL-22 and IL-17A, IL-22, IL-17A, and IFN-γ in TBL patients compared with those with PTB. This differential response was abrogated upon stimulation with anti-CD3 (Figure 3E). Thus, TBL is associated with a pathogen-specific expansion of CD4^+^ T cells expressing IL-22 and IL-17A, suggesting a role for these cells in pathogenesis.

### TBL is Associated with Decreased Frequencies of nTregs, which are Negatively Correlated with Baseline Frequencies of Mono- and Multifunctional Th1 Cells

Because TBL is associated with elevated frequencies of CD4^+^ Th1 and Th17 cells compared with PTB, we examined the absolute counts of CD4^+^ T cells as well as the frequencies of different CD4^+^ T cell subsets *ex vivo*. Our data reveal that TBL is not associated with significant alterations in CD4^+^ T cell numbers–as well as in the frequencies of naïve, central memory, and effector memory CD4^+^ T cells–in comparison to PTB (Table 1).

**Figure 4:**
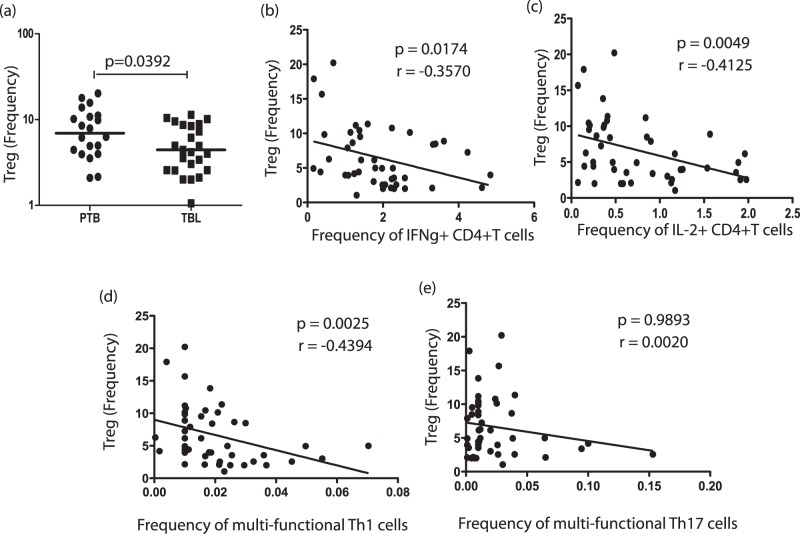
Tuberculous lymphadenitis (TBL) is associated with decreased frequencies of natural regulatory T cells (nTregs), which exhibit a negative relationship with mono- and multifunctional Th1 cells in TB-infected individuals. (*A*) Percentages of nTregs (defined as CD4^+^CD25^+^Foxp3^+^CD127^dim^ by flow cytometry) in tuberculous lymphadenitis (TBL) and pulmonary TB (PTB) individuals. (*B–E*) *Ex vivo* frequencies of nTregs were correlated with the baseline frequency of CD4^+^ T cells expressing IFN-γ alone (*B*), IL-2 alone (*C*). IFN-γ, IL-2, and TNF-α (*D*), or IL-17A, IL-17F, and IFN-γ (*E*) in all TB-infected individuals (*n* = 45). *P* values were calculated using the Spearman rank correlation test.

Because nTregs have been shown to be associated with downregulation of Th1 responses in active TB infection [30], [31], we also examined the baseline frequency of nTregs in TBL and PTB. Interestingly, the nTreg population–defined as CD4^+^CD25^+^Foxp3^+^CD127^dim^ cells–was present at a significantly lower frequency in TBL compared with PTB (Figure 4A). Also, correlation analysis between the frequencies of CD4^+^ T cells expressing single, double, or triple cytokines at baseline with frequencies of nTregs revealed a significantly negative correlation between the frequencies of mono- and multifunctional Th1 cells and those of nTregs in individuals with TB disease. Thus, the baseline frequency of CD4^+^ T cells expressing IFN-γ IL-2 and IFN-γ IL-2 TNF-α was negatively correlated with nTreg frequencies (Figure 4, B–D). There was no significant correlation between the frequencies of mono- (data not shown) and multifunctional Th17 cells (Figure 4E) and nTreg frequencies in individuals with TB disease. Thus, our data suggest that decreased frequencies of nTregs may at least partially account for the enhanced CD4^+^ Th1 cytokine responses in TBL individuals.

## Discussion

The immunological mechanisms involved in the pathogenesis of human TB are poorly understood. Although it is known that CD4+ T cells producing IFN-g are essential for protective immunity to TB, it is very likely that antigen – specific multifunctional T cells characterized by the coordinated expression of multiple effector functions, including other inflammatory cytokines, chemokines and effector molecules contribute to full protection against TB. Studies in animal models first revealed a potential association of multifunctional Th1 cells with protective immunity to TB [18], but recent studies in humans assessing differences in multifunctional T cell cytokine production in active PTB (compared with latent TB) have shown increased antigen-specific multifunctional CD4^+^ T cells [21], [22], [32], decreased frequencies [19], [20], or no difference [33]. Moreover, IFN-γ^+^IL-2^+^ co-expressing CD4^+^ T cells have been implicated in protective immunity to TB [34], [35], while dominant TNF-α single-positive CD4^+^ T cells have been postulated to be diagnostic of TB disease [20]. Finally, multifunctional CD4^+^ and CD8^+^ T cells have also been described to be associated with mycobacterial load in individuals with PTB [19]. Other studies have failed to report a role for dual-functional T cells in extrapulmonary TB [26]. Thus, the role of true multifunctional T cells in TBL (the most common extrapulmonary manifestation of TB) has, until the present, been left unstudied.

Our findings from examining CD4^+^ T cell responses involving Th1 cytokines reveal two interesting features. First, baseline differences in the frequency of CD4^+^ Th1 cells of the mono- and multifunctional variety suggest that the homeostatic immune environment in TBL is fundamentally different from that in PTB and is characterized by expansion of Th1 CD4^+^ cells. This is not surprising, as multi-focal lymphadenitis is secondary to hematogenous spread of bacteria from a pulmonary focus, while in PTB, the bacteria are confined to the lungs and draining lymph nodes. Second, the expansion of mono- and multifunctional Th1 cells in TBL individuals is relatively pathogen specific, because the differences in Th1 frequency profiles between the two groups of TB-infected patients are almost completely abolished on stimulation with a polyclonal stimulus (anti-CD3). Our study thus reiterates an important role for Th1 cells in the pathogenesis of TB disease and suggests that the mere presence of increased frequencies of Th1 cells does not correlate with protection. Indeed, elevated frequencies of Th1 cells might actually reflect enhanced severity of disease or may reflect the high antigen load seen in TBL.

Although it is well recognized that CD4^+^ Th1 cells are critical in cellular responses to TB, it is also clear that these responses alone are not sufficient [9]. Th17 cells, defined by the production of IL-17A and IL-17F, have been shown to play a central role in mediating immunity to both extra- and intracellular bacteria, including Mtb [29], [36]. The frequency of Th17 cells was found to be significantly lower in active TB compared with latent infection, and suppression of the Th17 response is considered to be an important mechanism in development of active TB [37]. Similarly, a subset of CD4^+^ T cells that expresses IL-22 (distinct from Th17 cells) is also thought to play an important role in immunity to human TB infection [17], [27]. In addition, CD4^+^ T cells expressing different combinations of IFN-γ, IL-2, TNF-α, IL-17A, and IL-22 have been described in tuberculous pleurisy [38], [39]. Finally, a recent study has reported that the CD4^+^ IFN-γ^+^IL-17A^+^ lymphocytes in peripheral blood and pleural fluid of TB patients correlated positively with clinical parameters associated with disease severity [25].

Our data on the examination of multifunctional Th17 cells reveal that Th17 cells expressing IL-17A and IL-17F as well as IL-17A, IL-17F, and IFN-γ are detectable in TB infection. This is the first study, to our knowledge, to demonstrate expression of both IL-17A and IL-17F in the same CD4^+^ T cell in a human infection. Similar to Th1 cells, mono- and multifunctional Th17 cells are present at higher frequencies both at baseline and following antigen stimulation in TBL individuals. Therefore, our study reveals a potentially important role for a multifunctional subset of Th17 cells in active TB and also suggests that CD4^+^ T cells expressing IL-17F alone might not play a critical role in TBL. While Th17 subsets have been previously reported in humans [24], our study is the first to demonstrate an antigen – specific response of these subsets in TB. Our study also examined regulation of classical Th22 cells (defined as IL-22^+^IL-17A^–^IFN-γ) to distinguish them from classical Th1 or Th17 cells) in TBL. Although Th22 cells have been postulated to play important roles in both (PTB and extrapulmonary TB [17], [38], we did not observe any significant alteration in the frequency of these cells either at baseline or following mycobacterial antigen stimulation in TBL individuals compared with those with PTB, suggesting that Th22 cells in the periphery are not key players in pathogenesis of TBL. However, our study also revealed a novel multifunctional subset of CD4^+^ T cells expressing IL-22 and IL-17A or IL-22, IL-17A and IFN-γ, which were specifically induced at higher frequencies in TBL individuals. The fact that the frequency of these cells was not altered at baseline or following anti-CD3 stimulation suggests that induction of this CD4^+^ T cell subset might be antigen specific and of potential importance in TBL pathogenesis.

One potential explanation for the increased baseline as well as antigen-specific expansion of CD4^+^ Th1 and Th17 cells could be increased CD4^+^ T cell total counts, altered subset distribution, or increased baseline activation of CD4^+^ T cells in TBL individuals. Our data reveal, however, that neither the total CD4^+^ T cell counts nor the proportion of naïve and memory CD4^+^ T cell subsets was significantly altered in TBL individuals, excluding the possibility that T cell numbers or subset distribution was the primary cause for the enhanced expansion of cytokine-producing T cells. Moreover, expression of Ki-67 (a marker of cycling)–which is a measure of activation/recent proliferation–was also not significantly different between the two groups at baseline (data not shown), suggesting that intrinsic differences in CD4^+^ T cell activation status might not be responsible for differential CD4^+^ T cell cytokine profiles. Therefore, TBL–although clinically a more disseminated form of TB disease–is not associated with alterations in the numbers, phenotype, or activation status of CD4^+^ T cells. Despite robust innate and adaptive immune responses, bacterial persistence is a key feature of TB infections, and Mtb is known to induce a variety of immunoregulatory mechanisms to resist elimination [40]. One such mechanism described in TB infections is induction of nTregs [41], which have been described to play a role in downregulating effector immune responses both at the site of infection and in the systemic circulation [30], [31], [42], [43]. Our data reveal that, at baseline, TBL individuals exhibit a significantly lower frequency of nTregs, which exhibit a significantly negative correlation with mono- and multifunctional Th1 (but not Th17) cells. Although we have not examined the functional activity of these cells or their response to antigenic stimuli, this correlation analysis clearly indicates that the deficiency of nTregs in TBL could at least partially account for the heightened expansion of pathogen-specific Th1 cells, although the exact mechanism is not known. This is in agreement with previous reports that nTregs in TB infections can selectively modulate IFN-γ- but not IL-17-expressing CD4^+^ T cells [25].

TB lymphadenitis is the commonest form of extrapulmonary TB and, in the face of HIV/AIDS, is becoming a major health problem worldwide [2]. In addition, TBL adds a layer of complexity in the field of TB due to the difficulty in diagnosis and treatment [3]. Our study systematically examines the CD4^+^ T cell responses in TBL and reveals an important role for CD4^+^ Th1 and Th17 cells in the pathogenesis of TBL. Our study also suggests that mono- or multifunctional CD4^+^ T cells in TB disease are not necessarily correlates of protection but may very well represent markers of disease severity and dissemination.
